# Pepfar 3.0’s HIV testing policy in Côte d'Ivoire (2014 to 2018): fragmentation, acceleration and disconnection

**DOI:** 10.1002/jia2.25424

**Published:** 2019-12-19

**Authors:** Anne Bekelynck, Joseph Larmarange, for the ANRS 12323 DOD‐CI Study Group, Nelly Assoumou, Anne Bekelynck, Christine Danel, Mohamed Doumbia, Mariatou Koné, Alexis Kouadio, Arsène Kra, Serge Niangoran, Honoré Ouantchi, Lazare Sika, Séverine Carillon, Maxime Inghels, Joseph Larmarange

**Affiliations:** ^1^ PAC‐CI/ANRS Research Site Program Treichville University Hospital Abidjan Ivory Coast; ^2^ Centre Population et Développement (Ceped) Université Paris Descartes, IRD, Inserm Paris France; ^3^ Institut de Recherche et Développement (IRD) Paris France

**Keywords:** Pepfar, HIV testing, Health policy, COP (Country operational Plan), Côte d'Ivoire, Africa

## Abstract

**Introduction:**

HIV Testing and Counselling (HTC) remains a key challenge in achieving control of the HIV epidemic by 2030. In the early 2010s, the President's Emergency Plan for AIDS Relief (Pepfar) adopted targeted HTC strategies for populations and geographical areas most affected by HIV. We examine how Pepfar defined targeted HTC in Côte d'Ivoire, a country with a mixed HIV epidemic, after a decade of expanding HTC services.

**Methods:**

We explored the evolution of HTC strategies through the Country Operational Plans (COP) of Pepfar during its phase 3.0, from COP 14 to COP 17 (October 2014 to September 2018) in Côte d'Ivoire. We conducted an analysis of the grey literature over the period 2014 to 2018 (Budget & Target Report, Strategic Direction Summary, Sustainability Index and Dashboard Summary, https://data.pepfar.gov). We also conducted a qualitative study in Côte d'Ivoire (2015 to 2018) using in‐depth interviews with stakeholders in the AIDS public response: CDC/Pepfar (3), Ministry of Health (3), intermediary NGOs (7); and public meeting observations (14).

**Results:**

Since the COP 14, Pepfar's HIV testing strategies have been characterized by significant variations in terms of numerical, geographical and population targets. While the aim of COP 14 and COP 15 seemed to be the improvement of testing efficacy in general and testing yield in particular, COP 16 and COP 17 prioritized accelerating progress towards the “first 90” (i.e. reducing the proportion of people living with HIV who are unaware of their HIV). A shift was observed in the definition of testing targets, with less focus on the inclusion of programmatic data and feedback from field actors, and greater emphasis on the use of models to estimate and disaggregate the targets by geographical units and sub‐populations (even if the availability of data by this disaggregation was limited or uncertain); increasingly leading to gaps between targets and results.

**Conclusions:**

These trials and tribulations question the real and long‐term effectiveness of annually‐revised, fragmented strategies, which widen an increasing disparity between the realities of the actors on the ground and the objectives set in Washington.

## 1 Introduction

HIV Testing and Counselling (HTC) remains the first challenge in achieving control of the HIV epidemic by 2030. Despite significant progress, it was estimated that in 2017 only 75% of people living with HIV (PLHIV) knew their status worldwide; this proportion dropping to 48% in western and central Africa 1. HTC is the gateway to accessing antiretroviral treatment, with individual benefits in terms of reduced rates of morbidity and mortality 2, as well as collective prevention benefits through the reduction in transmission risks stemming from the suppression of viral load among PLHIV on ART 3, 4.

Thanks to the emergence of international donors (in particular the US *President's Emergency Plan for AIDS Relief* or Pepfar in 2003 and the Global Fund to Fight AIDS, Tuberculosis and Malaria in 2002), HTC programmes have been scaled‐up across sub‐Saharan Africa.

Côte d'Ivoire was one of the 15 countries initially chosen by Pepfar in 2004, when adult HIV prevalence was 7%, and the only country in western Africa to receive the majority (72%) of its HIV funding from this donor, well ahead of the proportion contributed by the Global Fund to Fight AIDS Tuberculosis and Malaria (17%) and the Ivoirian State (11%) (2015 to 2017) 5. The HIV epidemic in Côte d'Ivoire is mixed, with a relatively low HIV prevalence in general population (2.9% in 2017 to 2018) 6 and significantly higher prevalence observed in some “key populations” such as female sex workers (12.2%) and men having sex with men (12.3%) (Unaids 2017).

In Côte d'Ivoire, Pepfar supported a set of measures that has considerably extended the coverage of people knowing their HIV status over the 2004 to 2014 period 7. Free HTC was adopted in 2004. HTC services have been gradually expanded to medical centres, tuberculosis sites and antenatal clinics. In 2010, the country adopted provider‐initiated testing and counselling strategies 8, 9, which recommend HTC to all adults and adolescents seen in all health facilities. Pepfar also implemented other strategies: mass HTC campaigns coupled with awareness campaigns, mobile and door‐to‐door HTC, and outreach activities for high‐risk groups. Although HTC coverage increased significantly (from 45,000 HIV tests performed in 2004 to more than 1.5 million in 2014), it was estimated that 87% of new infections originated from people unaware of their status over the period 2005 to 2015 10 and that only 54% of PLHIV knew their status in 2018 11.

Since the early 2010s, and the emerging hope for an “AIDS‐free generation” 12, 13, international guidelines have focused on intensifying efforts and accelerating the response to HIV. In 2011, the United Nations (UN) General Assembly adopted the *Political Declaration on HIV and AIDS: Intensifying Our Efforts to Eliminate HIV and AIDS* 14. In 2014, Unaids introduced the *90‐90‐90 targets – An ambitious treatment target to help end the AIDS epidemic* so that 90% of people living with HIV should know their status, 90% of those should be on treatment and 90% of those should have a controlled viral load by 2020 15. In June 2016, the UN General Assembly adopted its *Fast Track* strategy, with the aim of ending the HIV epidemic by 2030 16. These ambitious goals posed huge challenges to the capacity of health systems 17 and to the constrained resources available 18, 19.

In 2018, the UN stated that national and international resource availability for the global HIV response was far from the commitment made in the 2016 *Political Declaration* (approximately USD 19 billion instead of the USD 26 billion expected per year); and that funding levels had plateaued in recent years 20. With the increase in the number of people on treatment and the ever‐increasing needs, donors have faced strong pressure to optimize their resources 21.

In particular, Pepfar, which has been the largest bilateral donor in the fight against HIV, expressed its will to achieve a greater and accelerated impact, through better use of data, evidence and monitoring 22, 23, 24, 25. In December 2014, Pepfar initiated its phase 3.0. (*Controlling the Epidemic: Delivering on the Promise of an AIDS‐free Generation*), which was “pivoting to a data‐driven approach that strategically targets geographic areas and populations where [they could] achieve the most impact for [their] investments” 26. Regarding HTC, Pepfar moved from a “scale‐up” to a “scale‐down” approach 27, 28, 29, 30, with the adoption of HTC activities targeting specific populations and geographical areas, potentially most affected by HIV.

This article aims to analyse this paradigm shift, including how Pepfar defined and implemented targeted HTC strategies in Côte d'Ivoire as part of Pepfar 3.0, after a decade of expanding HTC services. We analyse the construction of Pepfar's strategic choices, between, on the one hand, the willingness to achieve better “value for money” through targeting HTC services and, on the other hand, the willingness to reduce the gaps in the first 90 target.

## 2 Methods

We used two primary sources of data: (i) grey literature produced by Pepfar between 2014 and 2018 and (ii) a qualitative survey conducted between 2015 and 2018.

Pepfar's grey literature included *Country Operational Plans* (COP)*, Budget & Target Reports*, *Strategic Direction Summary*, *Sustainability Index and Dashboard Summary* (when they existed), as well as internal presentations from the National Pepfar Office, *Country/Regional Operational Plan Guidance*, *Annual Report to Congress*, *Pepfar Strategic Plan 3.0* and Côte d'Ivoire's HTC data at https://data.pepfar.gov.

We explored the evolution of Pepfar's HTC strategies in Côte d'Ivoire using COP 14 to COP 17 (October 2014 to September 2018) corresponding to the Pepfar 3.0 period. The COP is an annual work plan for the US government that serves as the basis for approval of annual US government bilateral HIV/AIDS funding in most countries (https://data.pepfar.gov/glossary). For each funded country, it sets out Pepfar's strategy for the following year (for instance, COP 14 decides what will be funded during the fiscal year 2015, that is, from September 2014 to October 2015); it sets the targets that each implementer will have to achieve, by geographical area and by sub‐population. We tabulate these data by Pepfar objectives (i.e. the number of people receiving testing and counselling services, the number of people newly diagnosed with HIV, and the testing yield, defined as the number of HIV diagnoses divided by the number of tests undertaken), the population and geographical targets, and the strategic activities, from COP 14 to COP 17.

We supplemented this documentary research with qualitative interviews conducted with Pepfar staff in Côte d'Ivoire (3), representatives from the Ivorian Ministry of Health (3) as well as directors, monitoring & evaluation managers, project managers and regional coordinators from seven implementing non‐governmental organizations (three of whom mainly delivered clinic‐based services, and four providing community‐based services). The interviews focused on participants’ perspectives of recent developments in HTC policies, how targets were defined, the relevance of these targets, the effects of these policy changes on their current practice and the challenges they encountered. With the verbal consent of the respondants, the interviews were recorded, transcribed and then anonymized. In case of refusal, written notes were taken during the interview. We also conducted observations of meetings between Pepfar, its partners and the Ministry of Health (e.g. presentations of the mid‐term results of COP 15, preparations for COP 17) (2) as well as meetings held by the HTC Department of the National HIV/AIDS Program of the Ministry of Health (12). The interview and observation data were coded with the aid of NVivo 11, and analysed using a thematic approach.

This research was embedded within the “Demand and Supply for HIV and Hepatitis B and C Testing” project (ANRS 12323 DOD‐CI), which received ethical approval from the National Research Ethics Committee of the Ministry of Health of Côte d'Ivoire on 5 May 2015 (N°019/MSLS/CNER‐dkn).

## 3 Results

### 3.1 COP 14 (October 2014 to September 2015): improving the yield

In April 2014, Deborah Birx, a promoter of evidence‐based policies, was nominated as the US Global AIDS Coordinator. This appointment was in line with the elaboration of the COP 14 (occurring in spring 2014) and the Pepfar 3.0 plan. It established a global strategy looking for improved yield and return on investment, through targeted actions with high impact. A detailed table of targets (“Budget & Targets”) was introduced, with a breakdown by sex, age and HIV status. The COP 14 established a “high‐yield testing strategy,” to maximize the number of new HIV diagnoses at a constant cost, thereby representing increased value for money, and a greater chance of controlling the epidemic. Based on a mix of epidemiological and programmatic data, this COP introduced a categorization of health regions as “high‐yield” regions (14 of the 19 health regions managed by Pepfar) and “low‐yield” regions (5/19), with differentiated targets according to these new categories. Moreover, Pepfar decided to no longer support HTC sites with “low” or “zero” yield (less than five new HIV diagnosis per year), representing at that time 39% of testing sites in Côte d'Ivoire. General population‐oriented HTC strategies were no longer funded by Pepfar, with the COP giving priority to strategies focusing on key populations (female sex workers and men having sex with men) and priority populations (pregnant women, 15 to 24 year‐old women, 35 to 49 year‐old men, truckers and seasonal workers).

### 3.2 COP 15 (October 2015 to September 2016): rationalizing resources

**Figure 1:**
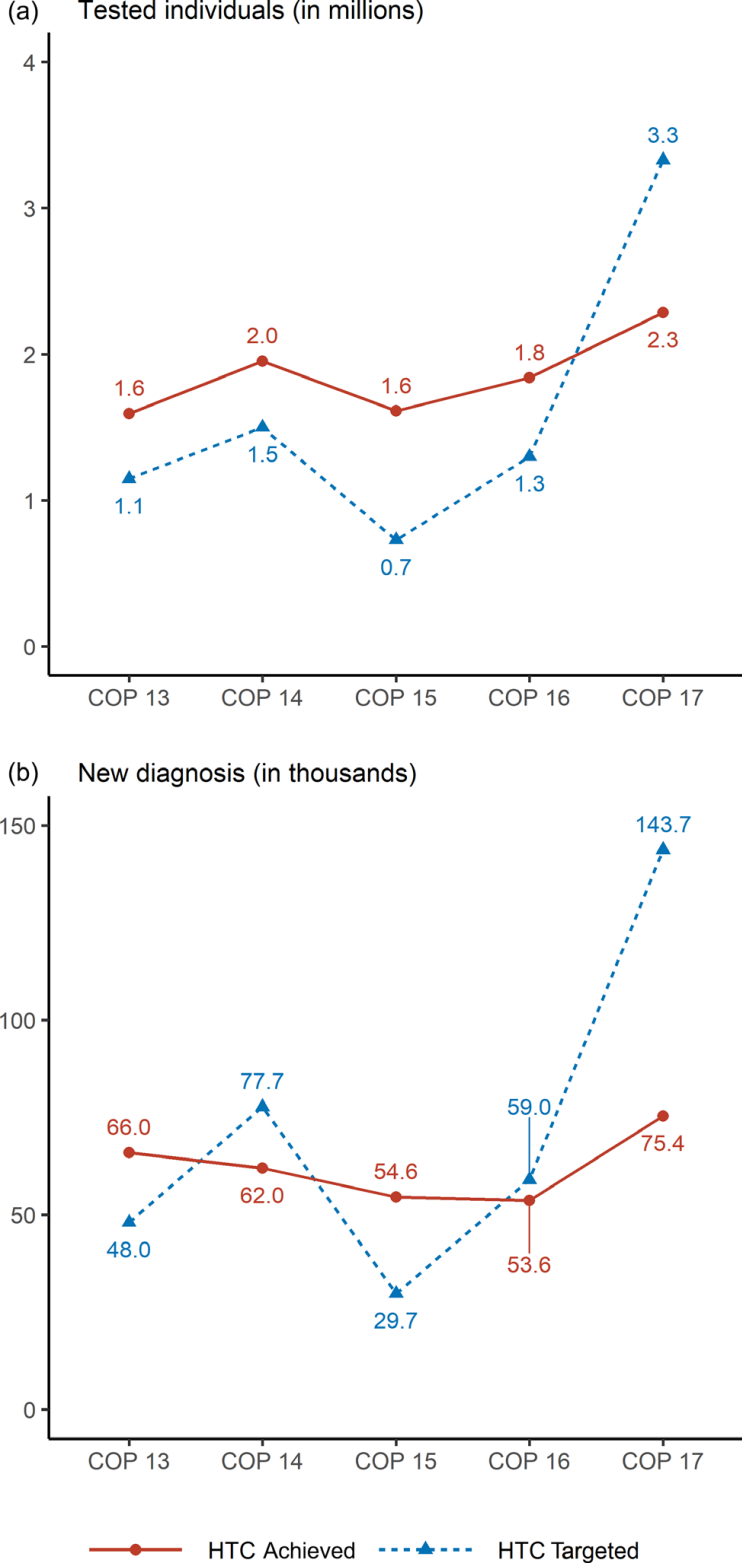
Targets and Results of Pepfar‐funded HIV testing activities in Côte d'Ivoire (COP 13–COP 17): **(a)** tested individuals and **(b)** new diagnoses.

The COP 15 (drafted in April 2015) was a step forward in promoting high impact strategies. Pepfar focused on rationalizing resources, and on ending what was perceived as a waste of inputs. The yield indicator was clearly presented in the COP 15 as a performance measure and the objectives in terms of new HIV diagnoses were finely disaggregated by sex, age and HTC approach. In view of their longer‐term strategy of preparing for their withdrawal, and their consideration that the country should finance a higher part of the HIV programme, Pepfar cut its budget allocated to HTC by 64%. In line with new Unaids estimates regarding the number of people living with HIV in Côte d'Ivoire in 2014 (370,000 31 compared to 450,000 32 in the previous estimate from 2013), Pepfar reduced by half the number of HIV tests to be funded through the COP 15 (Figure 1).

Geographical prioritization was refined at the finer scale of health districts (79 covered by Pepfar), distinguishing between the “maintenance/sustainable” districts (40 districts where the HTC offer was reduced to a minimum), the “scale‐up/aggressive scale‐up” (24 districts) and the “scale‐up to saturation/saturation” districts (15 districts where HTC was “active” or “massive”). National authorities did not officially adopt targeted HTC, but the weakness of their contribution to the budget of the national HIV response did not allow them to maintain, in practice, the principles of equity and of universal access to HTC, officially supported by the Ministry of Health 9.

While the COP 15 aimed to reduce the number of HIV tests performed, the number of individuals effectively tested for HIV was, at the end of the year, twice the target and represented only a small reduction compared to the number of tests conducted the previous year (Figure 1).

### 3.3 COP 16 (October 2016 to September 2017): reintroducing HTC as a priority

For the COP 16, HTC was reintroduced as a priority. The amount allocated to HTC increased from 2% to 6% of the total budget. Targets were re‐evaluated upward, with a doubling of the number of new HIV diagnoses to be achieved. This increase was again based on new estimates of the epidemic in Côte d'Ivoire from Unaids, the number of people living with HIV being now estimated at 460,000 33. This change was also in line with the introduction of the *Data Pack* system by the *Office of the US Global AIDS Coordinator* (OGAC) of Pepfar, used to define targets. Until then, the targets were (globally) defined in an inductive way, that is, according to past programmatic results (except for the COP 15 where a political choice prevailed). From Cop 16, Pepfar plans defined its targets in order to address the gap of the “1^st^ 90,” at the health district level, favoring greater variations from one year to the next and less flexibility for local actors.

### 3.4 COP 17 (October 2017 to September 2018): accelerating the search for HIV positives

The COP 17 marked a move to prioritize and intensify efforts on HTC, in line with all the debates on accelerating the control of the epidemic, formalized at the highest level in the *UN General Assembly Political Declaration on HIV and AIDS* adopted in June 2016 16. This translated into ambitious HTC targets: the number of new HIV diagnoses to be achieved in one year was multiplied by 2.4 (from 59,000 to 144,000). The detailed methodology used to produce this target was not available in the funder's grey literature. According to a Pepfar staff member in Côte d'Ivoire, this change was due to the literal application of the Unaids “1^st^ 90” target, especially since the country had committed itself to achieving it by 2020. For the COP 17, the objectives were defined in a deductive way via the *DataPack system*, so that 90% of the 460,000 people living with HIV in Côte d'Ivoire would know their status by 2020.

In order to accelerate progress towards the “1^st^ 90,” Pepfar adopted a two‐prong approach. First, it amended the characteristics of priority populations so as to increase the number of people to be targeted by testing services. In COP 17, men over 25 years old in the general population represented a new priority, recently defined by Unaids as a “blind spot” of the epidemic 34. Strategies promoted by Unaids and WHO in 2017 to reach these hard‐to‐reach populations 35 were being developed, such as index‐based testing (i.e. testing partners of people known to be living with HIV) and the introduction of self‐testing for key populations and men. Secondly, it developed risk assessment tools for HTC providers to use to screen persons, as for key populations and the families of Orphans and Vulnerable Children (OVC).

#### 3.4.1 Focus on the yield (COP 14 – COP 17)

**Figure 2:**
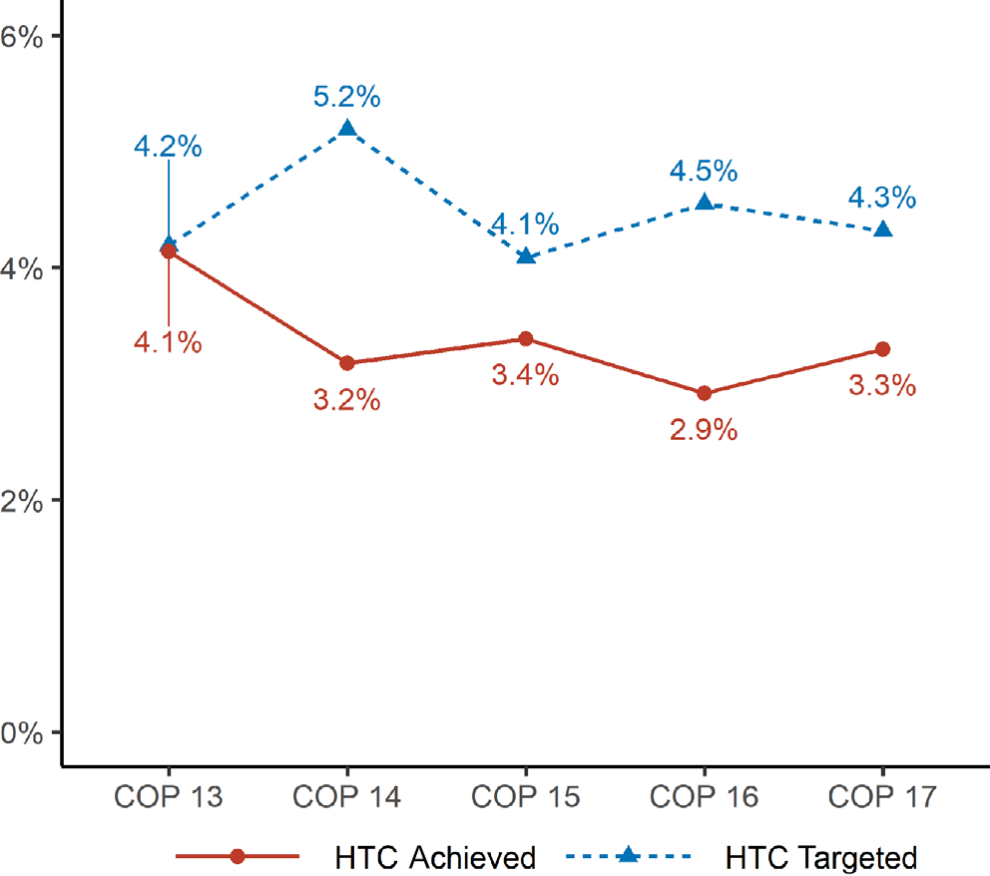
Yield of Pepfar‐funded HIV testing activities in Côte d'Ivoire (COP 13–COP 17) (in %).

Since COP 14, the yield of HIV testing has become an increasingly important indicator of testing performance. Although yield targets fluctuated between COP 14 and COP 17, they were systematically higher than the actual yields achieved by implementing partners which decreased (Figure 2).

## 4 Discussion

**Figure 3:**
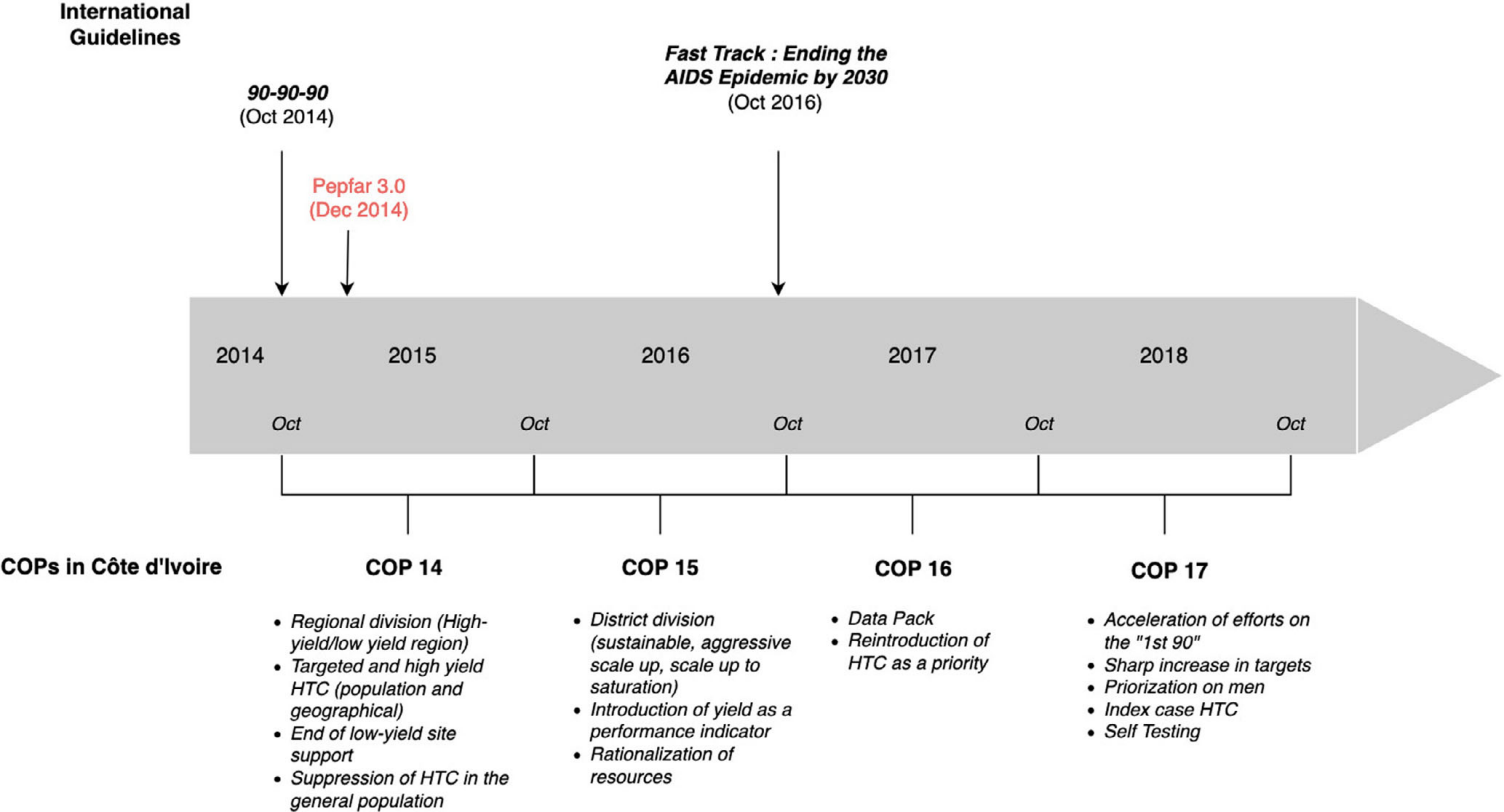
Timeline of Pepfar's HTC strategies in Côte d'Ivoire (COP 14 ‐ COP 17).

This analysis of the evolution of the Pepfar COPs in Côte d'Ivoire in recent years highlights how their approach significantly changed almost every year (Figure 3).

We observed significant variations in terms of numerical HTC targets, which were significantly reduced in COP 15, before becoming a programmatic priority in COPs 16 and 17. These different disruptions generated an increasingly pronounced gap between the targets that were set and the results that were achieved (see Figure 1), as implementers were not able to adapt their activities quickly enough to the shifting priorities.

Geographical targeting has also undergone significant changes, with a regional breakdown initially adopted (COP 14), followed by disaggregation at the health district level (COP 15), before being relatively stable (COP 16 to COP 17). These changes generated a reorganization of the implementing NGOs in the field, with human, technical and financial costs (readjustment of the actors of the national health system to the specific working methods of each implementing NGO, creation of trust between implementing NGOs, medical staff and local administrative authorities, redeployment of human resources). Targeted populations have also been subject to significant changes, with a focus on key populations and a few priority populations (COP 14), before expanding the scope to define men over 25 years as a new priority population.

The frequency and speed of these strategic reorientations were denounced by implementing NGOs as an obstacle to their efficiency and effectiveness, as they did not have time to adjust their strategies within the available timeframe.

“When you go through the first, second, third… when you get to the fourth and fifth places, you use less fuel because you have reached your cruising speed. We have trouble reaching our cruising speed, we spend our time downshifting, accelerating…” (Clinical NGO, Strategic Information Director)

This study underlines the limited possibility of implementing very rapidly evolving strategies – emphasized by the annual system of COPs – in a context where local actors have different adaptive capacities. Pepfar focused mainly on the rapid deployment of its strategies, to report quickly on visible results to the US Congress, so as to prove the effectiveness and legitimacy of this Presidential Plan 36, 37.

Our results also show the way in which Pepfar was paying increasing and demanding attention to the achievement of quantified and precise objectives. In this regard, a Pepfar representative said:

“I believe in the tyranny of averages. I don't like lumping countrywide data or even province‐wide data together. You lose the amazing positive deviance that provides insight into innovative solutions that are key to delivering services and improving quality. Data have to be accessible, and granular by age and gender so you have a clear understanding of who you are reaching, how you are reaching them, and what is working.” [in 38].

This statement is problematic at three levels.

First, this discourse on the supremacy of numbers contrasts with the uncertain nature of epidemiological estimates. The successive re‐estimates of the number of PLHIV in Côte d'Ivoire made by Unaids, and on which Pepfar bases its targets, contributed to significant variations in testing targets. Estimation methods are refined over time, making it possible to get closer to epidemiological realities; although each re‐estimation reveals past approximations and errors 39.

Second, the increasingly fine disaggregation required by the donor (by district, age, gender, status, etc.) contrasted with the lack of quality epidemiological data 25 available both at the district level and in relation to the size, prevalence and geographical location of key populations. The Population‐Based HIV Impact Assessment (PHIA) survey of Côte d'Ivoire, funded by Pepfar, was conducted in 2017 (three years after the first geographical breakdown) to provide more accurate data. Despite the size of the survey (approximately 10,000 households), HIV prevalence estimates could not be produced at the district level.

Third, these discourses on “accessible” data was not in line with the opacity of the methodology and data used by Pepfar to define its objectives 40. While the disaggregation of targets was increasingly detailed in the various COPs, no narrative explained the methodology for achieving it. For implementing NGOs, this methodology was often perceived as opaque, with no direct link to the realities they experienced in the field: “We don't know anything at all! When objectives arrive, it is barely if you know where they come from” (clinical NGO, Executive Director). A gap was widening between decision makers and those responsible for implementation.

Finally, whereas higher testing yield strategies are considered as being better value for money as they lead to more new HIV diagnoses for the same amount of money spent on testing, the results suggest failure, as yield has decreased over time. However, declining yields also reflect the fact that the easiest to reach people living with HIV have already been diagnosed and the remaining undiagnosed population is fewer and require more efforts. The significant resources deployed on improving testing yields have made testing approaches more complex to implement (e.g. requiring the development of detailed maps, implementation of risk assessment tools before HTC is proposed, refusal to screen “off‐target” individuals wishing to know their status). The focus on testing yield can undermine progress towards the first 90 in a context where most undiagnosed PLHIV are in the general population. Placing importance on quantified evaluations of activities can become counterproductive, by limiting the time dedicated to conducting testing activities 41, or because local implementers adopt strategies to bypass them 42.

From 2014 to 2018, it appeared that Pepfar adopted a trial and error approach. On one side, the financial crisis situation in the early 2010s led initially to the implementation of strategies focused on rationalizing resources and the need to develop high‐yield HTC (COPs 14 and 15). On the other side, the objective of achieving the 90‐90‐90 by 2020 and the “Fast Track” has emphasized the need to rapidly expand coverage of HTC, focusing on increasing the number of new HIV diagnoses (COPs 16 and 17).

## 5 Conclusions

The implementation of targeted HTC through Pepfar 3.0 was characterized by its fragmentation, acceleration and disconnection from the services delivering HTC, due to various factors: the annual and noncommittal COP system that persists despite the longevity of the Pepfar; alignment of programs with objectives based on imperfect data with continuous ongoing readjustments; and the absence of clearly identified HTC testing approaches in the Ivorian context of a mixed epidemic, oscillating between rationing resources and expanding HTC coverage. These trials and tribulations raised the question of the real and long‐term effectiveness of annually‐revised strategies which widen an increasingly pronounced gap between the realities of the implementing actors on the ground and the objectives set in Washington.

## Competing interests

Authors declare no competing interests.

## Authors’ contributions

AB conducted the research and wrote the first draft of the manuscript. JL contributed to the interpretation and presentation of the findings. All authors approved the final version of the manuscript for submission.

## Abbreviations

COP, Country Operational Plan; HTC, HIV Testing and Counselling; NGO, Non‐Governmental Organization; Pepfar, President's Emergency Plan for AIDS Relief; Unaids, Joint United Nations Programme on HIV/AIDS.
